# Efficacy of Oral Dextrose Gel for Neonatal Hypoglycaemia: An Updated Systematic Review and Meta‐Analysis of Randomized Controlled Trials With 4368 Patients

**DOI:** 10.1002/edm2.70327

**Published:** 2026-09-18

**Authors:** Rowa Tareq Al‐Enezi, Fatmah A. Alshebeeb, Julia Rose Panikulam, Dana B. AlKhaldi, Rawan B. Almutairi, Riyam Tareq Alenezi, Anwar M. Aljassar, Raghad B. Aldosari, Fajer Ali Alkandari, Amnah A. Dashti, Khadeja A. Aldhahi, Hamza A. Abdul‐Hafez, Abdullah M. Alharran

**Affiliations:** ^1^ Kuwait Institute for Medical Specializations Kuwait City Kuwait; ^2^ Tbilisi State Medical University Tbilisi Georgia; ^3^ Faculty of Medicine Kuwait University Kuwait City Kuwait; ^4^ Faculty of Medicine Alexandria University Alexandria Egypt; ^5^ Faculty of Medicine An‐Najah National University Nablus Palestine; ^6^ College of Medicine and Medical Sciences Arabian Gulf University Manama Kingdom of Bahrain

**Keywords:** dextrose gel, meta‐analysis, neonatal hypoglycaemia, newborn, NICU admission

## Abstract

**Background:**

Neonatal hypoglycaemia is a common metabolic disorder affecting up to 15% of healthy neonates and a higher proportion of at‐risk infants. Oral 40% dextrose gel has emerged as a promising, low‐cost intervention, but recent evidence remains inconsistent. This updated meta‐analysis evaluated the efficacy of 40% oral dextrose gel for preventing and treating neonatal hypoglycaemia.

**Methods:**

We systematically searched PubMed, Embase, Web of Science, Scopus and the Cochrane Library from inception to March 2026 for randomized controlled trials evaluating 40% oral dextrose gel in neonates at risk for or with confirmed hypoglycaemia. The primary outcome was hypoglycaemia. We used R software (version 4.5.0) for the analysis.

**Results:**

Eight RCTs comprising 4368 neonates were included. Dextrose gel did not significantly reduce hypoglycaemia, treatment success, recurrent hypoglycaemia, neonatal intensive care unit (NICU) admission, breastfeeding at discharge or formula feeding. Sensitivity analysis excluding Harding et al. (2021) for NICU admission yielded a statistically significant 24% reduction. Trim‐and‐fill adjustment for recurrent hypoglycaemia and formula feeding did not alter conclusions.

**Conclusions:**

Current evidence did not demonstrate a statistically significant reduction in overall hypoglycaemia, treatment failure, recurrent hypoglycaemia, NICU admission or breastfeeding interruption after administration of oral 40% dextrose compared with control. While safe and low‐cost, current evidence does not support routine use as a standalone intervention. Heterogeneity across protocols and populations warrants standardization in future trials.

**Trial Registration:**

PROSPERO registration number: CRD420261368171

## Graphical abstract

The efficacy of oral dextrose gel for neonatal hypoglycaemia.

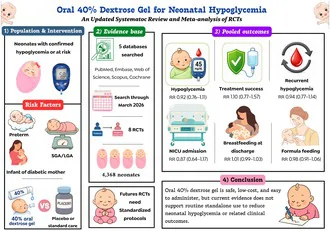

## 1 Introduction

Neonatal hypoglycaemia is a common metabolic disorder in the early neonatal period. It affects up to 15% of healthy neonates and a higher proportion of those with risk factors [1, 2]. A previous study reported an incidence of approximately 51% among high‐risk neonates [1, 2, 3]. The risk factors for neonatal hypoglycaemia include prematurity, small or large for gestational age and maternal diabetes [3]. Many healthy neonates experience a transient drop in glucose levels during the first few hours after delivery while adapting to extrauterine life [4]. Careful monitoring of neonatal hypoglycaemia is essential, considering its adverse neurodevelopmental effects [5]. Severe hypoglycaemia is associated with an increased risk of abnormal fine motor function, visual‐motor integration deficits and attention‐deficit/hyperactivity disorder symptoms [6]. Thus, early prevention and management of neonatal hypoglycaemia are essential.

The current management strategies for neonatal hypoglycaemia include early identification of high‐risk neonates, early feeding, close glucose monitoring and administration of intravenous (IV) dextrose in symptomatic or severe hypoglycaemia [7]. However, IV dextrose is invasive and requires neonatal intensive care unit (NICU) admission. Neonatal hypoglycaemia is a major cause of admission to the NICU, which is associated with increased costs, maternal–infant separation, maternal anxiety and breastfeeding interruption [8].

Oral 40% dextrose gel has emerged as a promising intervention for preventing and treating neonatal hypoglycaemia [7]. It is inexpensive, easy to administer via the buccal mucosa and could rapidly correct hypoglycaemia without interrupting breastfeeding [9, 10]. Thus, it could reduce NICU admission due to hypoglycaemia [11]. The landmark Sugar Babies trial first demonstrated that 40% dextrose gel reduced treatment failure compared with placebo in at‐risk late preterm and term infants with neonatal hypoglycaemia, with fewer infants admitted to the NICU for hypoglycaemia and reduced formula feeding at 2 weeks [11]. The larger hPOD multicentre trial confirmed a modest reduction in hypoglycaemia incidence but found no reduction in NICU admission [10].

A recent systematic review and meta‐analysis found that 40% oral dextrose gel significantly lowered admission to the NICU due to hypoglycaemia [12]. However, a recent study found no significant difference in admission to the NICU [13]. Other recently published studies found no significant difference between oral dextrose gel and the control group in treatment success defined as not requiring IV dextrose [14, 15]. In contrast, another trial suggested a benefit of oral dextrose gel in maintaining blood glucose levels [16].

Despite the growing body of evidence, important uncertainties persist. Prior meta‐analyses included few studies, and the evidence base has expanded substantially with the publication of several new randomized controlled trials, increasing the total sample size by approximately 1000 participants. Furthermore, existing syntheses have not comprehensively evaluated the full spectrum of clinically relevant outcomes across both prevention and treatment trial designs. The present meta‐analysis was therefore conducted to provide an updated and comprehensive synthesis of all available randomized evidence evaluating 40% dextrose gel for the prevention and treatment of neonatal hypoglycaemia.

## 2 Methods

### 2.1 Study Registration

The study protocol was registered with PROSPERO. This systematic review adhered to the Preferred Reporting Items for Systematic Reviews and Meta‐Analyses (PRISMA) 2020 statement standards [17]. The PRISMA checklist is provided in Supporting Information.

### 2.2 Literature Search and Study Selection

We searched PubMed, Embase, Web of Science, Scopus and Cochrane Library databases from inception up to March 2026 using the following search terms in PubMed: ((“Hypoglycemia”[Mesh] OR hypoglycem*[tiab] OR hypoglycaem*[tiab] OR “low blood glucose”[tiab] OR “low glucose”[tiab]) AND (“Infant, Newborn”[Mesh] OR neonat*[tiab] OR newborn*[tiab] OR “new born”[tiab] OR “new‐born”[tiab] OR infant*[tiab] OR baby[tiab] OR babies[tiab] OR preterm[tiab] OR premature[tiab]) AND (((“Dextrose”[Mesh] OR “Glucose”[Mesh] OR dextrose[tiab] OR glucose[tiab]) AND (gel[tiab] OR gels[tiab] OR oral[tiab] OR buccal[tiab] OR mucosal[tiab])) OR “oral dextrose gel”[tiab] OR “oral glucose gel”[tiab] OR “buccal dextrose gel”[tiab] OR “buccal glucose gel”[tiab] OR “40% dextrose gel”[tiab] OR “40% glucose gel”[tiab])) AND (randomized controlled trial[pt] OR controlled clinical trial[pt] OR random*[tiab] OR placebo[tiab] OR trial[tiab] OR groups[tiab]) NOT (animals[mh] NOT humans[mh]). Detailed search strategies are displayed in Table S1. Two independent reviewers conducted title/abstract screening followed by full‐text screening for final eligibility. Any disagreement was resolved by a third author's decision.

### 2.3 Eligibility Criteria

We included studies that met the following PICO criteria:

- Population: Neonates with confirmed hypoglycaemia (e.g., blood glucose < 2.6 mmol/L) or at risk (e.g., infants of diabetic mothers, preterm, large/small for gestational age).
- Intervention: Oral dextrose gel 40% administered for treatment or prophylaxis.
- Control: Placebo or standard care alone (e.g., feeding support as needed).
- Outcomes: At least one of the following: hypoglycaemia, treatment success (avoiding IV dextrose), recurrent hypoglycaemia, NICU admission, breastfeeding at discharge, formula feeding.
- Study design: Randomized controlled trials (RCTs) were included.

We excluded previous reviews, observational studies, cross‐sectional studies, single‐arm studies, case reports, case series, conference abstracts, finite element studies, in vitro studies, animal studies, editorials, non‐English studies and non‐peer‐reviewed studies.

### 2.4 Data Extraction

Two independent authors conducted the data extraction using a standardized Excel sheet. The standardized sheet included three domains:

1. Study characteristics including study design, registration number, country, sample size, population, inclusion criteria, intervention, control and primary outcomes.
2. Baseline characteristics of the included study population including number of participants, gestational age, birth weight, maternal data, sex and risk factors (maternal diabetes, preterm, SGA/LGA).
3. Measurements of the studied outcomes

### 2.5 Quality Assessment and Certainty of Evidence

Two independent authors assessed the risk of bias in included RCTs using the Cochrane Risk of Bias 2 (ROB2) tool, evaluating five domains: randomization process, deviations from intended interventions, missing outcome data, measurement of the outcome and selection of reported results [18]. Conflicts were resolved by a third author. Results were visualized using the RobVis web tool [19]. Certainty of evidence was assessed using the Grading of Recommendations Assessment, Development, and Evaluation (GRADE) framework [20].

### 2.6 Statistical Analysis

All statistical analyses were performed using R software (version 4.5.0; R Foundation for Statistical Computing, Vienna, Austria) with the meta, metasens, readxl and tidyverse packages [21]. For dichotomous outcomes (hypoglycaemia, treatment success, recurrent hypoglycaemia, NICU admission, breastfeeding and formula feeding), pooled effect estimates were calculated using the risk ratio (RR) with corresponding 95% confidence intervals (CIs). The meta‐analysis of hypoglycaemia included only prevention trials with outcome not present at baseline, while treatment success included treatment trials. The meta‐analysis was conducted using the inverse variance method within a random‐effects model to account for between‐study heterogeneity. A random‐effects model (restricted maximum likelihood estimator, REML) was applied in all analyses to account for between‐study variability. Statistical heterogeneity was assessed using the *I* ^2^ statistic, Cochran's *Q* test and *τ* ^2^, with *I* ^2^ values interpreted as low (< 25%), moderate (25%–75%) or high (> 75%) heterogeneity.

Sensitivity analysis was performed using a leave‐one‐out approach, sequentially omitting each study and recalculating the pooled effect estimate to assess robustness. In cases where heterogeneity remained substantial despite leave‐one‐out analysis, Baujat plots were constructed to identify studies contributing disproportionately to overall heterogeneity and effect size influence. Subgroup analysis was conducted according to study purpose as either prevention or treatment, with *p* < 0.05 indicating statistically significant interaction. Publication bias and small‐study effects were assessed according to the number of included studies. For outcomes including fewer than 10 studies, the Doi plot and the Luis Furuya–Kanamori (LFK) index were used [22]. If major asymmetry was detected, the trim‐and‐fill method was applied to adjust for potential missing studies [23].

## 3 Results

### 3.1 Literature Search

**FIGURE 1:**
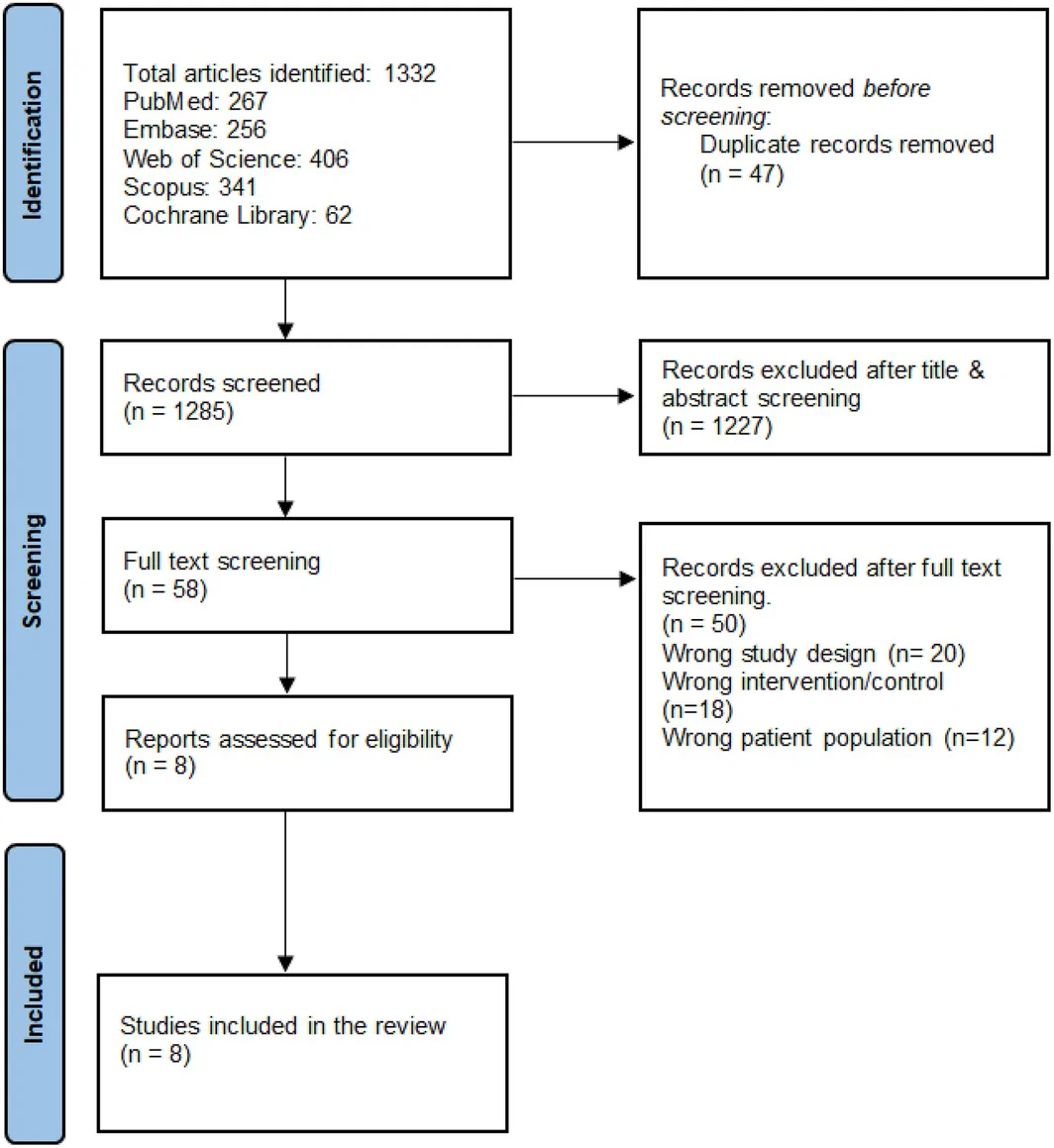
PRISMA flow chart for the systematic search and selection process.

Our literature search initially retrieved 1332 records. After removing duplicates, 1285 studies were screened by title and abstract. Of these, 58 were reviewed in full‐text screening. Finally, eight publications from eight original RCTs met eligibility criteria and were included [10, 11, 13, 14, 15, 16, 24, 25]. The PRISMA flow diagram is shown in Figure 1.

### 3.2 Study Characteristics

A total of 8 RCTs encompassing 4368 participants were included [10, 11, 13, 14, 15, 16, 24, 25]. The number of patients included in our analysis was 4368: 2256 neonates in the 40% dextrose gel group and 2112 neonates in the control group. The included studies varied in population (preterm, late preterm, small for gestational age (SGA), large for gestational age (LGA), infants of diabetic mothers), intervention protocols (prophylactic vs. treatment) and control groups (placebo gel, breastfeeding, formula feeding). Detailed summaries of included studies and baseline characteristics are reported in Tables 1 and 2.

**TABLE 1:** Summary characteristics of the included studies.

Study ID	Country	Trial registration	Design	Prevention/treatment	Setting	Population	Intervention	Comparator	Randomized *N* (I)	Randomized *N* (C)	Hypoglycaemia threshold	Primary outcome
Covas et al. (2023) [15]	Argentina	NR	Randomized non‐inferiority clinical trial (open‐label)	Treatment	Private hospital, rooming‐in setting	At‐risk neonates with early asymptomatic hypoglycaemia	40% glucose gel 0.5 mL/kg buccal	Formula milk 9 mL/kg	118	123	< 35 mg/dL at 2 h and < 47 mg/dL thereafter	Therapeutic failure (persistent/recurrent hypoglycaemia in first 48 h)
Gupta et al. (2022) [24]	India	CTRI/2017/11/010383	Stratified randomized controlled trial (open‐label)	Treatment	Tertiary centre	At‐risk infants with asymptomatic hypoglycaemia	40% dextrose gel + breastfeeding	Breastfeeding alone	147	144	< 25, 35, 45 mg/dL corresponding to 0–4, 4–24, 24–48 h, respectively	Treatment failure/need for IV fluids
Harris et al. (Sugar Babies) (2013) [11]	New Zealand	ACTRN12608000623392	Randomized, double‐blind, placebo‐controlled trial	Treatment	Single tertiary centre	At‐risk late preterm/term infants (35–42 weeks), < 48 h old, with hypoglycaemia	40% oral dextrose gel 200 mg/kg + feeding	Placebo gel + feeding	118	119	< 2.6 mmol/L	Treatment failure (< 2.6 mmol/L after two treatment attempts)
Hegarty et al. (Pre‐hPOD) (2016) [10]	New Zealand	ACTRN12613000322730	Randomized, double‐blind, placebo‐controlled dose‐finding trial	Prevention	Two hospitals	At‐risk neonates without NICU indication	Any prophylactic 40% dextrose gel dose (single or multiple; 200–1000 mg/kg total)	Any matching placebo gel dose	277	138	< 2.6 mmol/L	Hypoglycaemia < 2.6 mmol/L in first 48 h
Harding et al. (hPOD) (2021) [25]	New Zealand and Australia	ACTRN12614001263684	Multicentre randomized, double‐blind, placebo‐controlled trial	Prevention	18 maternity hospitals	At‐risk neonates without indication for NICU admission	0.5 mL/kg 40% dextrose gel at 1 h of age	Identical placebo gel	1070	1063	< 2.6 mmol/L	NICU admission > 4 h
King et al. (GEHPPI) (2025) [13]	Ireland and Czech Republic	NCT04353713	Randomized, blinded, placebo‐controlled trial	Prevention	Four level‐3 and one level‐2 neonatal units	Inborn infants ≤ 32 + 0 weeks GA in delivery room	40% dextrose gel in delivery room (0.5 mL if ≤ 29 + 0 weeks; 1 mL if ≥ 29 + 1 weeks)	Placebo gel	84	85	< 1.8 mmol/L	Hypoglycaemia < 1.8 mmol/L at first IV access on NICU admission
Pandurangan and Bhosgi (2025) [14]	India	CTRI/2024/03/064726	Randomized controlled trial (open‐label)	Prevention	Tertiary care centre	At‐risk neonates (LGA, SGA, IDM, IUGR, preterm < 35 weeks)	40% oral dextrose gel after birth + breastfeeding	Breastfeeding alone	26	26	< 45 mg/dL	Incidence of hypoglycaemia
Polo Perucchin et al. (2025) [16]	Italy	NR	Two‐phase randomized trial/pilot (not blinded)	Prevention	Single centre	At‐risk infants in Phase 1; late preterm infants only in Phase 2	Early prophylactic 40% dextrose gel at 15 min of life	No dextrose gel	100 + 50	100 + 50	Moderate 25–40 mg/dL, Severe < 25 mg/dL	Blood glucose/moderate hypoglycaemia in first hours after birth

Abbreviations: BGL, blood glucose level; DG, dextrose gel; h, hour(s); IQR, interquartile range; mg/dL, milligrams per decilitre; min, minute(s); mmol/L, millimoles per litre; *n*, number; NICU, neonatal intensive care unit; NR, not reported; ODG, oral dextrose gel; RCT, randomized controlled trial; SD, standard deviation; vs., versus.

**TABLE 2:** Baseline characteristics of the participants.

Study ID	Arm	Dose/regimen	Analysed *N*	Maternal age	Maternal diabetes/DM	Gestational age	Birth weight	Sex (female), *n* (%)	Delivery mode	Singleton/multiples	Baseline/early glucose	Risk profile	Other baseline details
Covas et al. (2023) [15]	Glucose gel 40%	40% glucose gel 0.5 mL/kg buccal	107	NR	6 infants of diabetic mothers	35 weeks:1; 36 weeks:14; 37 weeks:10; 38 weeks:32; 39 weeks:26; 40 weeks:22; 41 weeks:2	3399.28 g mean	57 (25.7%)	NR	NR	Eligibility based on early asymptomatic hypoglycaemia	PEG 23; GEG 63; IDM 6; late preterm 15	Analysed *n* = 107 after excluding symptomatic/withdrawn participants
	Formula milk	Formula milk 9 mL/kg	115	NR	4 infants of diabetic mothers	35 weeks:0; 36 weeks:20; 37 weeks:14; 38 weeks:36; 39 weeks:32; 40 weeks:12; 41 weeks:1	3440.93 g mean	56 (25.2%)	NR	NR	Eligibility based on early asymptomatic hypoglycaemia	PEG 26; GEG 65; IDM 4; late preterm 20	Analysed *n* = 115 after post‐randomization exclusions
Gupta et al. (2022) [24]	Dextrose gel	40% dextrose gel + breastfeeding	147	29.2 ± 4.56 years	68 (46.3%) gestational diabetes	Late preterm > 35 weeks: 55 (37.4%)	2802 ± 618 g	70 (47.6%)	51 caesarean (34.7%)	NR	0–4 h median 22 (21–24) mg/dL	SGA/IUGR 56 (38.0%); IDM 68 (46.3%); late preterm 55 (37.4%)	PIH 31 (21.1%); anaemia 34 (23.1%); multiple pregnancy 11 (7.4%)
	Control	Breastfeeding alone	144	29.5 ± 5.13 years	70 (48.6%) gestational diabetes	Late preterm > 35 weeks: 62 (43.0%)	2728 ± 573 g	68 (47.2%)	47 caesarean (32.6%)	NR	0–4 h median 22.3 (21–24.8) mg/dL	SGA/IUGR 55 (38.1%); IDM 70 (48.6%); late preterm 62 (43.0%)	PIH 35 (24.3%); anaemia 39 (27.0%); multiple pregnancy 14 (9.7%)
Harris et al. (Sugar Babies) (2013) [11]	Dextrose	40% dextrose gel 200 mg/kg + feeding	118	29.2 (6.0) years	46/115 (40%)	37.4 (1.6) weeks	3091 (824) g	70 (59.3%)	73 vaginal (62%)	100 (85%)	2.2 (1.1–2.5) mmol/L	IDM 46 (39%); late preterm 41 (35%); < 2500 g 30 (25%); > 4500 g 12 (10%); < 10th centile 13 (11%); > 90th centile 26 (22%)	Intended breastfeeding 114/115 (99%); NZ European 53%; Maori 29%
	Placebo	Placebo gel + feeding	119	30.2 (6.5) years	46/115 (40%)	37.2 (1.6) weeks	3031 (782) g	54 (45.4%)	74 vaginal (62%)	99 (83%)	2.2 (0.9–2.5) mmol/L	IDM 46 (39%); late preterm 49 (41%); < 2500 g 32 (27%); > 4500 g 10 (8%); < 10th centile 19 (16%); > 90th centile 27 (23%)	Intended breastfeeding 109/115 (95%); NZ European 54%; Maori 31%
Hegarty et al. (Pre‐hPOD) (2016) [10]	Any dextrose	Any prophylactic 40% dextrose gel dose (single or multiple)	277	32 (5) years	201/275 (73%)	38 (1) weeks	3244 (607) g	139 (50%)	127 caesarean (46%)	256 (92%)	NR	IDM 201 (73%); late preterm 21 (8%); small 31 (11%); large 24 (9%); two risk factors 6 (9%)	Birthweight z score 0 (1); NZ European 31%; Pacific 17%; Maori 8%
	Any placebo	Any matching placebo gel dose	138	32 (6) years	100/138 (72%)	38 (1) weeks	3248 (633) g	63 (46%)	72 caesarean (52%)	128 (93%)	NR	IDM 100 (72%); late preterm 6 (4%); small 18 (13%); large 14 (10%); two risk factors 4 (6%)	Birthweight z score 0 (1); NZ European 21%; Pacific 14%; Maori 14%
Harding et al. (hPOD) (2021) [25]	Dextrose	0.5 mL/kg 40% dextrose gel at 1 h of age	1070	32.2 (5.3) years	863/1070 infants of mothers with diabetes (80.7%)	38.4 (1.1) weeks	3328 (613) g	515 (48.1%)	436 caesarean (42.5%)	991 (92.7%)	Initial glucose 3.16 (0.77) mmol/L	IDM 863 (80.7%); preterm 75 (7.0%); small 83 (7.8%); large 49 (4.6%); two risk factors 175 (16.5%)	Maternal diabetes type: gestational 740 (88.6%), type 1 38 (4.6%), type 2 57 (6.8%)
	Placebo	Identical placebo gel	1063	32.2 (5.4) years	856/1063 infants of mothers with diabetes (80.5%)	38.5 (1.1) weeks	3313 (594) g	523 (49.2%)	405 caesarean (39.5%)	999 (94.3%)	Initial glucose 2.97 (0.69) mmol/L	IDM 856 (80.5%); preterm 76 (7.2%); small 84 (7.9%); large 47 (4.4%); two risk factors 178 (16.8%)	Maternal diabetes type: gestational 732 (88.3%), type 1 31 (3.7%), type 2 66 (8.0%)
King et al. (GEHPPI) (2025) [13]	Dextrose	40% dextrose DR gel (0.5 mL if ≤ 29 + 0 weeks; 1 mL if ≥ 29 + 1 weeks)	84	NR	Pre‐pregnancy 2 (2%); gestational 7 (8%)	29 + 1–32 + 0: 45 (54%); ≤ 29 + 0: 39 (46%)	1399 (308) g [29 + 1–32 + 0]; 898 (273) g [≤ 29 + 0]	41 (49%)	67 caesarean (80%)	Multiple pregnancy 29 (35%)	Primary outcome measured at median 54 min	Very/extreme preterm only	Complete antenatal steroids 67 (80%); MgSO4 73 (87%); age at gel 14.0 min
	Placebo	Placebo DR gel	85	NR	Pre‐pregnancy 2 (2%); gestational 10 (12%)	29 + 1–32 + 0: 48 (56%); ≤ 29 + 0: 37 (44%)	1473 (387) g [29 + 1–32 + 0]; 895 (310) g [≤ 29 + 0]	42 (49%)	64 caesarean (75%)	Multiple pregnancy 24 (28%)	Primary outcome measured at median 52 min	Very/extreme preterm only	Complete antenatal steroids 64 (75%); MgSO4 79 (93%); age at gel 12.7 min
Pandurangan and Bhosgi (2025) [14]	Dextrose	40% oral dextrose gel after birth + breastfeeding	26	NR	Gestational diabetes 4; overt DM 1	35 ± 1.4 weeks	2.2 ± 0.8 kg	NR	17 LSCS	NR	NR	LGA 4; SGA 6; IUGR 7; preterm < 35 weeks. 9; IDM 2	Gestational HTN 10; anaemia 6; pre‐eclampsia 4
	Control	Breastfeeding without oral dextrose gel	25	NR	Gestational diabetes 3; overt DM 0	36 ± 0.6 weeks	2.8 ± 1.2 kg	NR	20 LSCS	NR	NR	LGA 3; SGA 8; IUGR 8; preterm < 35 weeks 7; IDM 1	Gestational HTN 12; anaemia 8; pre‐eclampsia 3; 1 sepsis exclusion post‐randomization
Polo Perucchin et al. (2025) [16]	Dextrose (First phase)	40% dextrose gel at 15 min of life	100	NR	DMG 40	39.07 ± 1.51 weeks	3253.3 ± 693.3 g	45 (45%)	NR	NR	2‐h glucose 57.23 ± 15.93 mg/dL (post‐intervention measure)	SGA 31; LGA 31; DMG 40; GA < 37 weeks 12	Open‐label single‐centre first phase
	Control (First phase)	No dextrose gel	100	NR	DMG 30	38.66 ± 4.25 weeks	3173.8 ± 700.6 g	45 (45%)	NR	NR	2‐h glucose 57.72 ± 12.59 mg/dL (post‐assignment measure)	SGA 33; LGA 32; DMG 30; GA < 37 weeks. 17	8/16 infants with moderate hypoglycaemia later required IV glucose and were excluded from follow‐up
	Dextrose (Second phase)	40% dextrose gel at 15 min of life	50	NR	DMG 10	36 ± 0.7 weeks	2493 ± 398 g	NR	NR	NR	2‐h glucose 57.62 ± 17.97 mg/dL	LP cohort; SGA 5; LGA 4; DMG 10	Among infants without infusion, 2‐h glucose 58.48 ± 17.33 mg/dL
	Control (Second phase)	No dextrose gel	47	NR	DMG 9	36 ± 0.8 weeks	2523 ± 389 g	NR	NR	NR	2‐h glucose 52.94 ± 15.76 mg/dL	LP cohort; SGA 6; LGA 3; DMG 9	3 control neonates withdrawn in first hour due to clinical symptoms

Abbreviations: %, percentage; BGL, blood glucose level; BW, birth weight; GA, gestational age; GDM, gestational diabetes mellitus; IDM, infant of diabetic mother; IQR, interquartile range; LGA, large for gestational age; mg/dL, milligrams per decilitre; mmol/L, millimoles per litre; *n*, number; NR, not reported; SD, standard deviation; SGA, small for gestational age.

### 3.3 Quality Assessment and Certainty of Evidence

**FIGURE 2:**
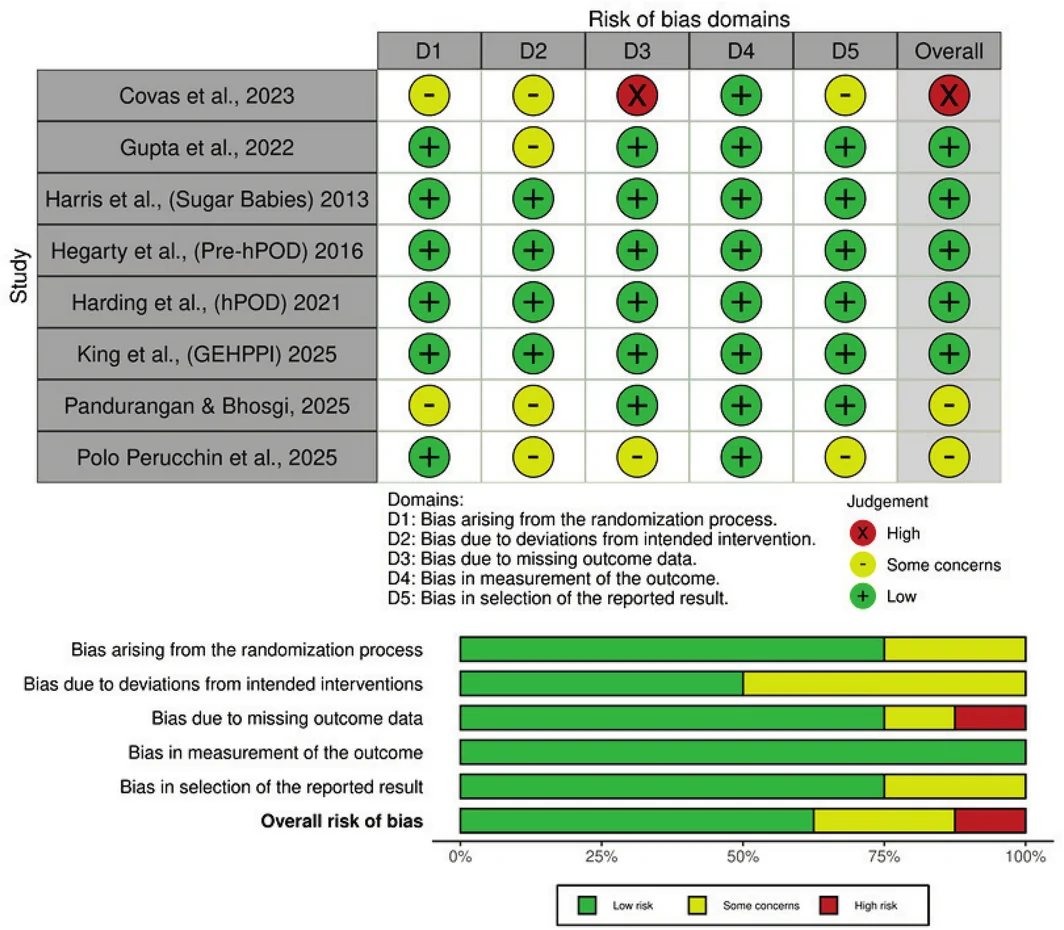
Summary and traffic light plots of the risk of bias of the included randomized controlled trials.

Eight original RCTs were evaluated using the ROB2 assessment tool. Five studies had an overall low risk of bias [10, 11, 13, 24, 25], two studies showed some concerns [14, 16], and one study had a high risk of bias [15] (Figure 2). GRADE assessment for major outcomes ranged from moderate to very low as presented in Table 3.

**TABLE 3:** Grading of Recommendations Assessment, Development, and Evaluation (GRADE) evidence.

Outcome	Number of studies	Study design	Risk of bias	Inconsistency	Indirectness	Imprecision	Publication bias	Effect estimate (95% CI)	Certainty
Hypoglycaemia	5	RCTs	Not serious	Serious (*I* ^2^ = 63.4%)	Not serious	Serious^b^	Not detected	RR 0.92 (0.76 to 1.11)	⨁⨁◯◯ Low
Treatment success	3	RCTs	Serious^a^	Serious (*I* ^2^ = 95%)	Not serious	Serious^b^	Not detected	RR 1.10 (0.77 to 1.57)	⨁◯◯◯ Very Low
Recurrent hypoglycaemia	3	RCTs	Not serious	Not serious (*I* ^2^ = 0%)	Not serious	Serious^b^	Major asymmetry (LFK = 5.38)	RR 0.94 (0.77 to 1.14)	⨁⨁◯◯ Low
NICU admission	3	RCTs	Not serious	Serious (*I* ^2^ = 61.3%)	Not serious	Serious^b^	Not detected	RR 0.87 (0.64 to 1.17)	⨁⨁◯◯ Low
Breastfeeding at discharge	3	RCTs	Not serious	Not serious (*I* ^2^ = 0%)	Not serious	Serious^b^	Minor asymmetry (LFK = 1.80)	RR 1.01 (0.99 to 1.03)	⨁⨁⨁◯ Moderate
Formula feeding	3	RCTs	Not serious	Not serious (*I* ^2^ = 0%)	Not serious	Serious^b^	Major asymmetry (LFK = −4.38)	RR 0.98 (0.91 to 1.06)	◯◯ Low

^a^ Risk of Bias, Serious: One study had high risk of bias (Covas et al. [15]). Downgraded by one level.

^b^ Imprecision, Serious: 95% confidence intervals cross the null (no effect) and are wide, indicating no statistically significant effect. The individual outcome meta‐analyses included only three studies, limiting precision. Downgraded by one level.

### 3.4 Outcomes

#### 3.4.1 Primary Outcome

##### 3.4.1.1 Hypoglycaemia

**FIGURE 3:**
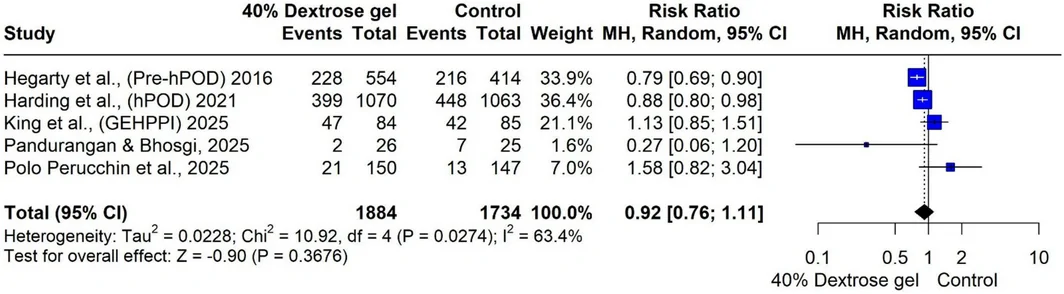
Forest plot of hypoglycaemia comparing 40% dextrose gel versus control.

The meta‐analysis of five prevention trials (*n* = 3618) showed no statistically significant difference and the pooled risk ratio (RR) was 0.92 (95% CI: 0.76 to 1.11; *p* = 0.37). There was moderate heterogeneity across studies (*I* ^2^ = 63.4%, *p* = 0.027) (Figure 3). Heterogeneity was not resolved with leave‐one‐out sensitivity analysis (Figure S1). Influence analysis demonstrated that no single study disproportionately affected the overall meta‐analysis. While Hegarty et al. (Pre‐hPOD) 2016 had the greatest influence on the effect size, its contribution to heterogeneity was relatively modest. In contrast, studies such as King et al. [13] (GEHPPI) and Polo Perucchin et al. [16] contributed more to heterogeneity but had limited influence on the overall result. Harding et al. [25] (hPOD) and Pandurangan and Bhosgi [14] showed minimal influence on both the pooled estimate and heterogeneity (Figure S2). The Doi plot showed a symmetrical distribution, with an LFK index of −0.48, indicating no evidence of publication bias or small‐study effects (Figure S3).

#### 3.4.2 Secondary Outcomes

##### 3.4.2.1 Treatment Success

**FIGURE 4:**
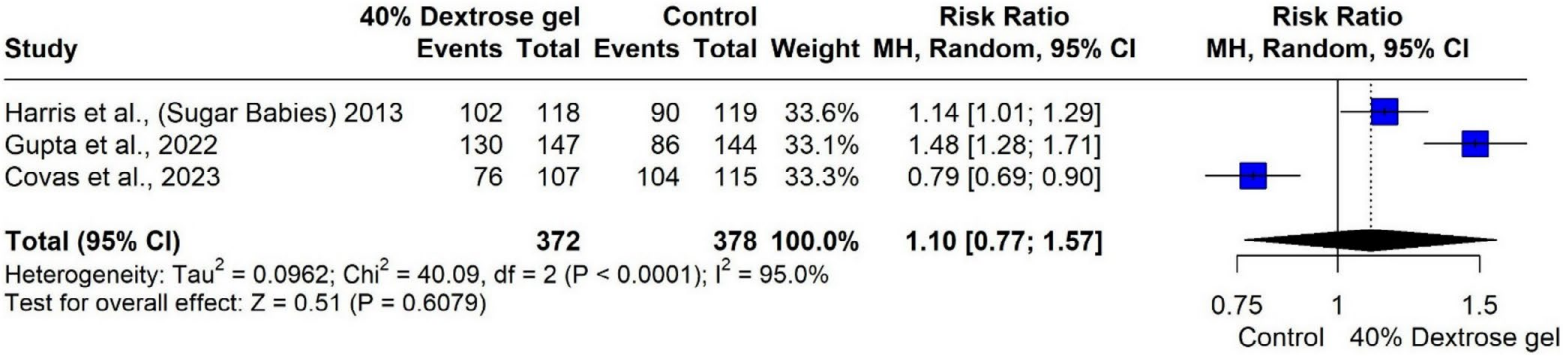
Forest plot of treatment success comparing 40% dextrose gel versus control.

The meta‐analysis of three studies (*n* = 750) showed no significant difference in treatment success between 40% dextrose gel and control (RR = 1.10, 95% CI: 0.77–1.57; *p* = 0.61). However, there was considerable heterogeneity among studies (*I* ^2^ = 95%), indicating substantial variability in results across trials (Figure 4), which was not resolved by leave‐one‐out sensitivity analysis (Figure S4). Influence analysis indicated that Gupta et al. [24] had the greatest impact on the overall effect estimate, while Covas et al. [15] contributed most to heterogeneity. Harris et al. [11] (Sugar Babies) showed minimal influence (Figure S5). The Doi plot showed no asymmetry (LFK index = −0.19), indicating no evidence of publication bias (Figure S6).

##### 3.4.2.2 Recurrent Hypoglycaemia

**FIGURE 5:**
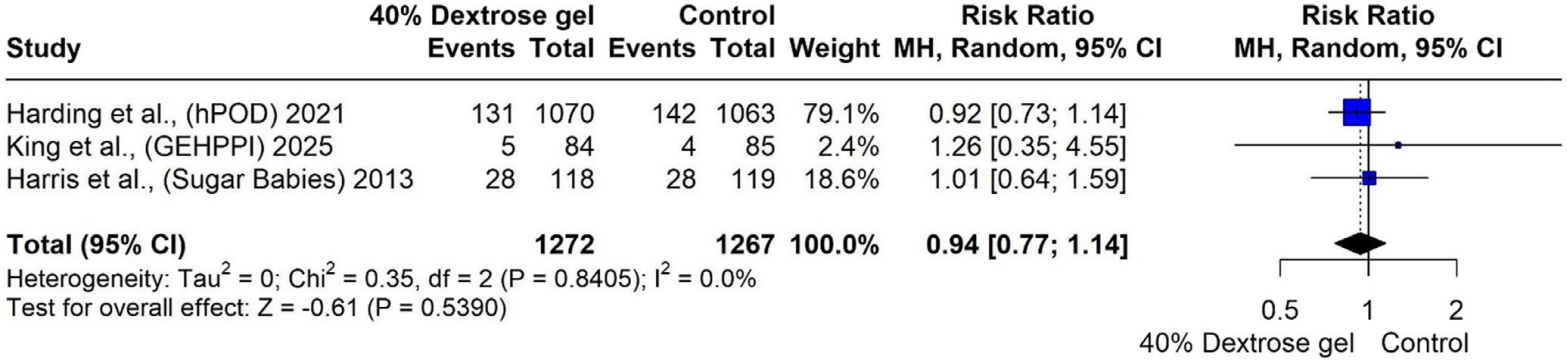
Forest plot of recurrent hypoglycaemia comparing 40% dextrose gel versus control.

The meta‐analysis of three studies (*n* = 2539) showed no significant difference in recurrent hypoglycaemia between 40% dextrose gel and control (RR = 0.94, 95% CI: 0.77–1.14; *p* = 0.54), with no observed heterogeneity (*I* ^2^ = 0%) (Figure 5). The Doi plot showed major asymmetry (LFK index = 5.38) (Figure S7). After applying the trim‐and‐fill method (adding two imputed studies), the adjusted analysis showed no significant difference between 40% dextrose gel and control (RR = 0.92, 95% CI: 0.77–1.10; *p* = 0.34). There was no evidence of heterogeneity (*I* ^2^ = 0%), and the results remained consistent with the primary analysis, suggesting that potential publication bias did not materially affect the overall findings (Figure S8).

##### 3.4.2.3 Admission to NICU

**FIGURE 6:**
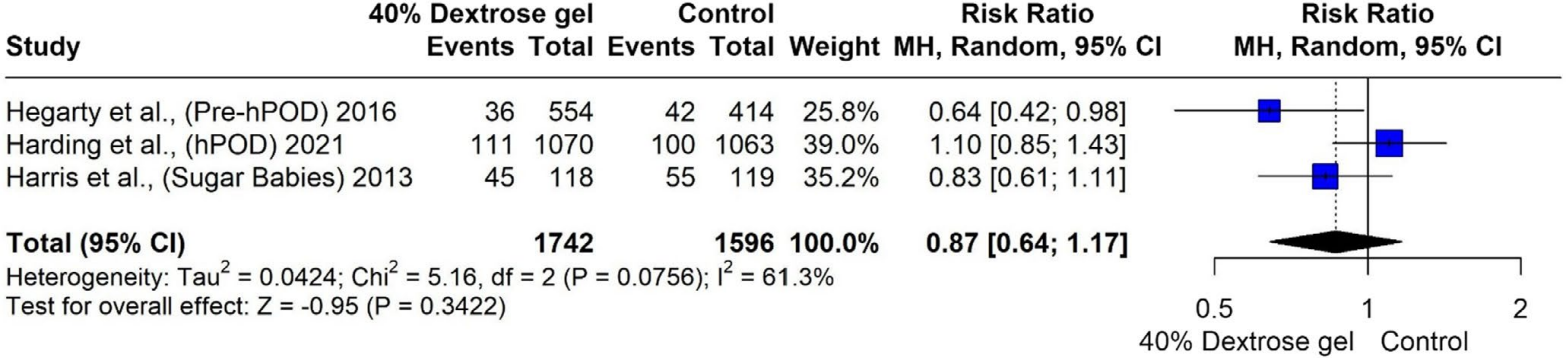
Forest plot of NICU admission comparing 40% dextrose gel versus control.

The meta‐analysis of three studies (*n* = 3338) showed no significant difference in NICU admission between 40% dextrose gel and control (RR = 0.87, 95% CI: 0.64–1.17; *p* = 0.34), with moderate heterogeneity (*I* ^2^ = 61.3%) (Figure 6). Heterogeneity was primarily attributable to Harding et al. [25]; excluding this study reduced *I* ^2^ to 0% and resulted in a statistically significant effect (RR = 0.76, 95% CI: 0.59–0.97) (Figure S9). The Doi plot showed no asymmetry (LFK index = −0.76) (Figure S10).

##### 3.4.2.4 Breastfeeding at Discharge

**FIGURE 7:**
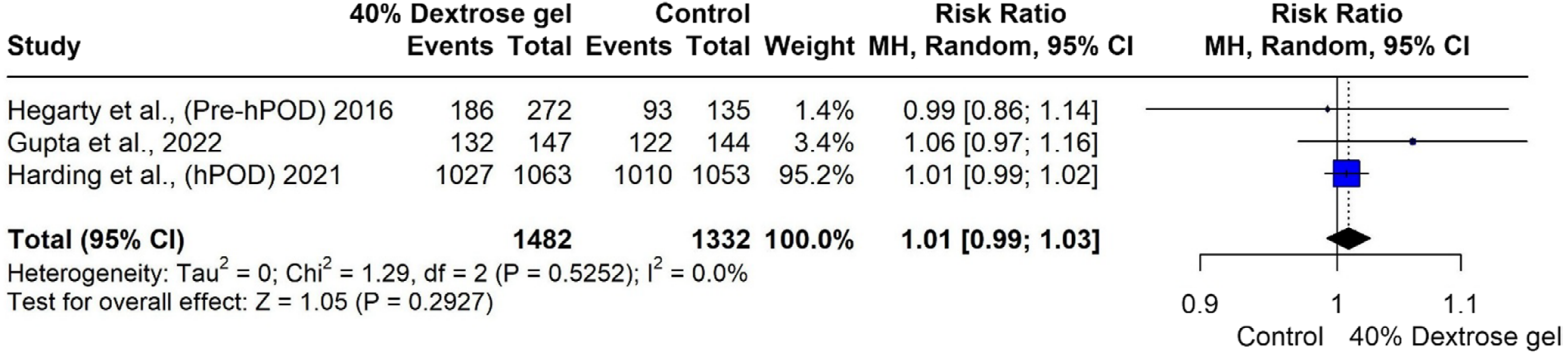
Forest plot of breastfeeding at discharge comparing 40% dextrose gel versus control.

The meta‐analysis of three studies (*n* = 2814) showed no significant difference in breastfeeding at discharge between 40% dextrose gel and control (RR = 1.01, 95% CI: 0.99–1.03; *p* = 0.29), with no heterogeneity (*I* ^2^ = 0%) (Figure 7). The Doi plot showed minor asymmetry (LFK index = 1.80) (Figure S11).

##### 3.4.2.5 Any Formula Feeding

**FIGURE 8:**
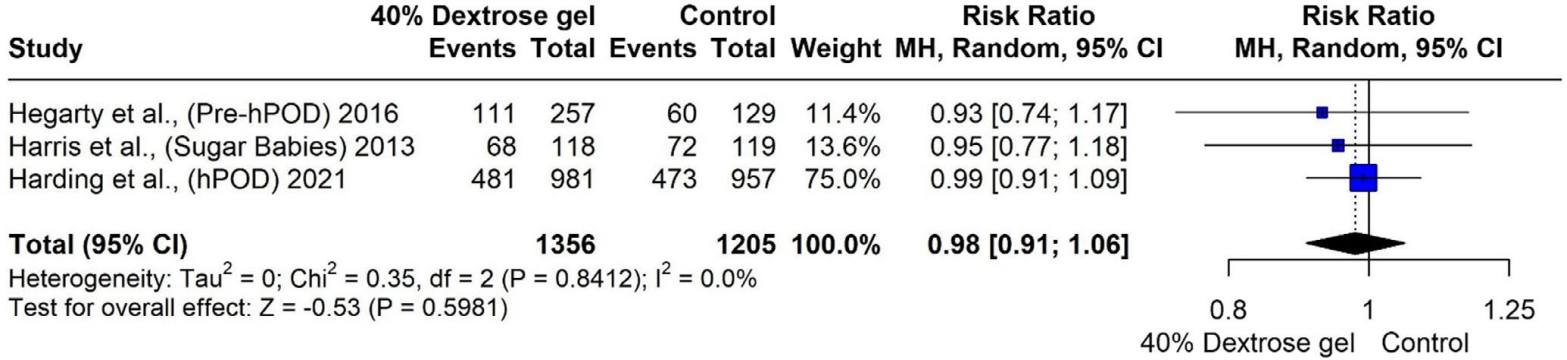
Forest plot of formula feeding comparing 40% dextrose gel versus control.

The meta‐analysis of three studies (*n* = 2561) showed no significant difference in formula feeding between 40% dextrose gel and control (RR = 0.98, 95% CI: 0.91–1.06; *p* = 0.60), with no heterogeneity (*I* ^2^ = 0%) (Figure 8). The Doi plot showed major asymmetry (LFK index = −4.38) (Figure S12). After applying the trim‐and‐fill method (adding two studies), the adjusted analysis showed no significant difference between 40% dextrose gel and control (RR = 0.99, 95% CI: 0.92–1.06; *p* = 0.82), with no heterogeneity (*I* ^2^ = 0%) (Figure S13).

#### 3.4.3 Subgroup Analysis

To determine whether treatment effects differed by trial purpose, we stratified analyses for outcomes with data from both prevention and treatment trials. Results were similarly non‐significant for recurrent hypoglycaemia, breastfeeding at discharge and formula feeding with no observed heterogeneity. For NICU admission, prevention trials showed substantial heterogeneity and had a pooled effect of (RR 0.86 (0.51–1.47); *I* ^2^ = 78.1%), while the single treatment trial yielded an RR of 0.83 (0.61–1.11). Tests for subgroup differences were not significant for any outcome (all *p* > 0.05), indicating that the current evidence is limited to draw definitive conclusions (Figures S14–S17).

## 4 Discussion

### 4.1 Summary of Findings

We investigated the efficacy of 40% oral dextrose gel for preventing and treating neonatal hypoglycaemia. Our systematic review and meta‐analysis included eight studies with 4368 participants. We found no significant difference between 40% dextrose and the control group in the prevention of hypoglycaemia, treatment success, hypoglycaemia recurrence, admission to the NICU, breastfeeding or any feeding. Importantly, these non‐significant findings should not be interpreted as evidence of equivalence between oral dextrose gel and standard care. The wide confidence intervals for several outcomes, particularly treatment success and NICU admission, include both clinically meaningful benefits and harms that could alter practice recommendations. These estimates are therefore inconclusive rather than definitive, and the current evidence is insufficient to rule out a clinically important effect of dextrose gel in either direction. Subgroup analyses stratified by trial purpose of either prevention or treatment yielded null results, with no evidence of differential efficacy between settings but with limited statistical power.

### 4.2 Interpretation of Findings

Hypoglycaemia occurs when there is an imbalance between glucose production and utilization demand [26]. This can occur due to reduced glucose stores (e.g., in preterm infants) or increased glucose demand (e.g., in infants of diabetic mothers) [26].

Oral 40% dextrose is suggested to reduce the incidence of hypoglycaemia, reducing the need for IV dextrose, avoiding mother‐infant separation and the potential adverse events of hyperglycemia that could occur with IV dextrose [27, 28, 29]. In our study, we found no significant difference in treatment success between neonates receiving oral dextrose gel and the control group. The 95% CI showed a possible 23% reduction to a 57% increase in treatment success. Covas et al. [15] found that treatment success was significantly lower in the dextrose groups (71%) compared with the control group (90.4%). This may be explained by the observation that formula‐fed newborns have higher glucose levels than breastfed neonates [30]. The neonates in the control group in Covas et al. used formula feeding. Harris et al. used a placebo gel and mainly breastfeeding as the control group, while Gupta et al. used breastfeeding in the control group [15, 24].

The available evidence suggests that the efficacy of dextrose gel may differ across neonatal subgroups though the evidence is inconsistent. The infant of diabetic mother (IDM) constituted 81% of the hPOD trial population [25] and a substantial proportion of other studies. In Gupta et al. [24], IDM/LGA infants showed significant benefit from dextrose gel (RR 0.31, 95% CI 0.14–0.66). They found that the treatment failure rate was significantly lower among IDM. Yet, Harding et al. [25] found no evidence of differential effect on performing subgroup analysis based on the risk factor. On the other hand, for late preterm infants, Polo Perucchin et al. [16] found benefit in late preterm infants, with significantly higher blood glucose levels in the dextrose gel group at 2 h. Furthermore, they observed benefit for SGA infants, suggesting that they may benefit from early glucose supplementation. Meanwhile, King et al. [13] found no benefit of dextrose gel in infants ≤ 32 weeks' gestation, with identical rates of hypoglycaemia in both groups (29%).

The Pre‐hPOD secondary analysis using continuous glucose monitoring confirmed that prophylactic dextrose gel reduced the risk of initial hypoglycaemia without increasing recurrent or severe episodes [10]. In our study, we found no difference in the incidence of recurrent hypoglycaemia. Notably, oral dextrose does not address the underlying metabolic disorder that leads to episode recurrence, such as inborn errors of metabolism or hyperinsulinism [11, 16, 31]. This could occur due to a hereditary error of metabolism or increased insulin secretion. In these cases, other treatments such as hydrocortisone or glucagon infusion could be used [31].

Our findings differ from the recent meta‐analysis by Sivakumar et al. [12], which reported a significant reduction in NICU admission with oral dextrose gel. This discrepancy likely reflects the inclusion of additional randomized trials in our analysis that contributed to the null findings for NICU admission. The exclusion of Harding et al. [25] in our sensitivity analysis reduced heterogeneity and yielded a significant 24% reduction in NICU admission. The 95% CI in our analysis showed a potential 36% reduction to a 17% increase in admission rates. This may be explained by differences in intervention protocols: one trial used a single prophylactic oral dose at the first hour, whereas others used multiple doses as needed [10, 11, 25]. The single dose may be insufficient to prevent NICU admission since many infants develop hypoglycaemia after the first few hours. Furthermore, the frequency, duration of glucose monitoring and thresholds were not specified in the trial protocol and differed across the trial sites [25]. This highlights the need for standardized dosing protocols in future research. Our meta‐analysis also extends previous work by providing comprehensive GRADE assessments for all major outcomes, subgroup analyses stratified by trial purpose and detailed evaluation of breastfeeding and feeding outcomes that were not systematically examined in prior syntheses.

Among the included studies, there was variation in delivery mode, control groups, included populations and timing of oral dextrose administration. This variability may have contributed to the inconsistent findings across studies. One study found that 40% oral dextrose had no significant effect on reducing hypoglycaemia in caesarean‐delivered neonates. Meanwhile, neonates that were vaginally delivered had significantly lower rates of hypoglycaemia [13]. On the other hand, no significant difference in the incidence of hypoglycaemia was reported after accounting for the mode of delivery [10].

Doses ranged from a single dose of 200 mg/kg [24, 25] to higher doses of 400 mg/kg [10] and multi‐dose regimens of up to 1000 mg/kg total [10]. Polo Perucchin et al. [16] used a fixed volume (1–2 mL) rather than weight‐based dosing. The lack of a dose–response effect suggests that higher doses may not confer additional benefit; yet this requires confirmation [10]. Timing varied from immediately after stabilization in the delivery room [13, 16] to 1 h after birth [10, 25] and up to 2 h after birth [15, 24]. Earlier administration may be more effective for prevention, but the optimal timing remains uncertain [16]. However, Giuseppe et al. [32] found that the timing of oral dextrose administration had no significant impact on the incidence of hypoglycaemia. Also, the comparators varied from placebo gel [10, 11, 13, 16], breastfeeding alone [14, 24] and formula feeding [15]. The choice of comparator is particularly important since formula feeding itself is an effective treatment for hypoglycaemia.

Definitions of hypoglycaemia had thresholds ranging from < 1.8 mmol/L [13] to < 2.6 mmol/L [10, 11, 25] and < 45 mg/dL [16, 24]. Higher thresholds may detect more events, potentially increasing statistical power but also including mild episodes which may be of little clinical significance. Meanwhile, lower thresholds detect severe episodes but may miss clinically relevant cases. Some studies used scheduled monitoring [10, 11], while others used pragmatic hospital protocols [13, 25]. This may have influenced the detection of hypoglycaemia and treatment effect. Diagnostic methods varied from glucose oxidase methods to bedside glucometers, which have known variability at low glucose concentrations. This variability introduces misclassification bias that attenuates effect estimates toward the null. Standardization of hypoglycaemia definitions in future trials would improve the interpretability of results.

A possible concern is that oral dextrose gel might affect breastfeeding. Several studies have shown that receiving any supplements in the neonatal period may delay breastfeeding initiation and reduce breastfeeding duration [33, 34, 35]. In our study, we found no significant difference in breastfeeding at discharge or formula use between gel and control groups. This suggests that oral dextrose gel does not impact breastfeeding. The included studies enroled mothers intending to breastfeed, with formula feeding used as part of hypoglycaemia protocols [11, 25].

The current data indicate that this breastfeeding benefit is likely contingent on whether dextrose gel successfully prevents NICU admission and maternal–infant separation, rather than being an intrinsic pharmacological effect of the gel itself. Clinicians should therefore counsel families that while dextrose gel is unlikely to harm breastfeeding, its ability to promote exclusive breastfeeding depends on the broader clinical trajectory. Specifically, its benefit depends on whether it prevents escalation to more invasive interventions that disrupt mother–infant bonding. No adverse events were reported in the included studies. However, long‐term safety requires further investigation, as neurodevelopmental impairment may not become apparent until later in life.

### 4.3 Strengths and Limitations

Our study has several strengths. We followed PRISMA guidelines and included only RCTs, representing high‐quality evidence. Our meta‐analysis investigated several outcomes, such as hypoglycaemia recurrence, need for IV dextrose and the effect on breastfeeding and feeding at discharge. We included large multicentre studies that were not included in earlier meta‐analysis [12]. These studies contributed to the total sample size, improving the precision and generalizability of the findings. We conducted leave‐one‐out sensitivity analyses, used Baujat plots to identify studies contributing disproportionately to heterogeneity and influence, and assessed publication bias using trim‐and‐fill methods. Assessment of certainty of evidence was conducted using the GRADE framework.

Several limitations warrant consideration. First, despite the large total sample size, individual outcome meta‐analyses included only 3–5 studies, making the analyses susceptible to Type II error and insufficient statistical power to detect modest but clinically important differences. The null findings for outcomes with only three studies should be interpreted as inconclusive rather than evidence of no effect. Second, substantial unexplained heterogeneity for some outcomes indicates that the pooled estimates may not apply directly to any single population. This likely reflects clinical and methodological diversity (dosing regimens, control group compositions, populations) and may have biased estimates toward the null.

Third, studies included clinically distinct neonatal populations with different baseline hypoglycaemia risks. While this enhances generalizability, it may obscure subgroup‐specific effects. We were unable to perform stratified analyses due to insufficient reporting of outcome data by risk category. Pooling prevention and treatment trials may have biased estimates toward the null if the intervention is more effective in one setting, and clinical diversity may have inflated *I* ^2^ values. Non‐significant subgroup differences suggest insufficient evidence to detect differential efficacy. Fourth, the definition of hypoglycaemia varied across studies, introducing non‐differential misclassification bias that typically attenuates effect estimates toward the null. Yet, this variability is reported in the literature [31]. Fifth, the included studies were conducted in high‐income countries limiting generalizability. There was variability in the used protocols; some studies used oral dextrose as prophylaxis, while others used it for the treatment of asymptomatic hypoglycaemia. Sixth, publication bias assessment was limited, as Doi plots, LFK indices and trim‐and‐fill analyses have limited power to detect bias with fewer than 10 studies. These methods are designed for larger meta‐analyses, and their results should be interpreted cautiously.

### 4.4 Clinical Implications and Future Directions

Our systematic review and meta‐analysis have provided implications for clinical practice. Although oral dextrose gel did not significantly reduce hypoglycaemia incidence or NICU admission, its potential safety, low cost and ease of administration suggest it may have a role as an adjunctive therapy, particularly in resource‐limited settings or where IV access is delayed. Dextrose gel may be used as a temporary measure while awaiting IV access or transfer to higher‐level care. It could also be incorporated into a multimodal strategy including early feeding, glucose monitoring and clear escalation protocols. It should not be used as a replacement for IV dextrose when clinically indicated. Although the existing evidence did not demonstrate a benefit when administering oral dextrose 40%, several implementation factors should be considered. For instance, staff training is essential to ensure correct administration technique and appropriate timing. Protocol standardization should specify indications for use, dosing, monitoring frequency and escalation criteria. Families should be counselled that dextrose gel is an adjunct to feeding not a replacement. Cost‐effectiveness data are limited, but Glasgow et al. [9] suggested potential cost savings. Real‐world effectiveness may differ from efficacy demonstrated in controlled trials, and regular auditing is needed to ensure that protocols are followed and outcomes are monitored.

The findings of our study should be interpreted with caution, considering the limited number of included studies and the significant heterogeneity in some outcomes. Several high‐priority research gaps remain. First, dose‐finding trials are urgently needed to determine whether multi‐dose regimens are superior to single‐dose prophylaxis. Second, population‐specific trials should stratify enrolment by hypoglycaemia mechanism (hyperinsulinism, glycogen depletion, prematurity) and report outcomes separately. Third, continuous glucose monitoring should replace intermittent glucose checks to provide area‐under‐the‐curve metrics. Finally, long‐term neurodevelopmental follow‐up is essential.

## 5 Conclusion

Current evidence did not demonstrate a significant benefit of oral 40% dextrose gel in reducing the overall incidence of neonatal hypoglycaemia compared with control. There were comparable rates of hypoglycaemia recurrence, treatment success, admission to the NICU and breastfeeding at discharge. However, these findings should be considered preliminary and interpreted with caution, given the limited number of included studies, substantial heterogeneity across protocols and the wide confidence intervals that include potentially clinically meaningful effects. Non‐significant results do not establish equivalence between dextrose gel and standard care. Future large‐scale, well‐blinded randomized trials with standardized hypoglycaemia definitions, dose‐finding protocols and long‐term neurodevelopmental follow‐up are warranted.

## Author Contributions

**Fatmah A. Alshebeeb:** data curation, writing – original draft. **Dana B. AlKhaldi:** data curation, writing – original draft. **Julia Rose Panikulam:** data curation, writing – original draft. **Fajer Ali Alkandari:** data curation, writing – original draft. **Rowa Tareq Al‐Enezi:** writing – original draft, methodology, data curation, writing – review and editing. **Rawan B. Almutairi:** data curation, writing – original draft. **Raghad B. Aldosari:** data curation, writing – original draft. **Amnah A. Dashti:** data curation, writing – original draft. **Khadeja A. Aldhahi:** data curation, writing – original draft. **Riyam Tareq Alenezi:** data curation, writing – original draft. **Anwar M. Aljassar:** data curation, writing – original draft. **Abdullah M. Alharran:** methodology, formal analysis, writing – original draft, writing – review and editing, supervision. **Hamza A. Abdul‐Hafez:** data curation, writing – original draft.

## Funding

The authors have nothing to report.

## Disclosure

Artificial Intelligence: The authors report that no artificial intelligence tools were used in any stage of the study.

## Ethics Statement

The authors have nothing to report.

## Consent

The authors have nothing to report.

## Conflicts of Interest

The authors declare no conflicts of interest.

## Supporting information


**Data S1:** PRISMA checklist.
**Table S1:** Search strategies.
**Figure S1:** Leave‐one‐out sensitivity analysis for hypoglycaemia.
**Figure S2:** Baujat plot for hypoglycaemia.
**Figure S3:** Doi plot for hypoglycaemia.
**Figure S4:** Leave‐one‐out sensitivity analysis for treatment success.
**Figure S5:** Baujat plot for treatment success.
**Figure S6:** Doi plot for treatment success.
**Figure S7:** Doi plot for recurrent hypoglycaemia.
**Figure S8:** Funnel plot after trim and fill meta‐analysis for recurrent hypoglycaemia.
**Figure S9:** Leave‐one‐out sensitivity analysis for NICU admission.
**Figure S10:** Doi plot for NICU admission.
**Figure S11:** Doi plot for breastfeeding at discharge.
**Figure S12:** Doi plot for formula feeding.
**Figure S13:** Funnel plot after trim and fill meta‐analysis for NICU admission.
**Figure S14:** Forest plot of recurrent hypoglycaemia by trial purpose.
**Figure S15:** Forest plot of breastfeeding at discharge by trial purpose.
**Figure S16:** Forest plot of formula feeding by trial purpose.
**Figure S17:** Forest plot of NICU admission by trial purpose.

## Data Availability

The data that support the findings of this study are available from the corresponding author upon reasonable request.
