# Supplementary material for: Efficacy of Oral Dextrose Gel for Neonatal Hypoglycaemia: An Updated Systematic Review and Meta‐Analysis of Randomized Controlled Trials With 4368 Patients
Source: Endocrinol Diabetes Metab. 2026 Sep 18;9(5):e70327. doi: 10.1002/edm2.70327 (PMC13587362; doi:10.1002/edm2.70327)
Supplement: Supplementary file 1 [file EDM2-9-e70327-s001.docx]

**Supplementary Appendix**

**Table of Contents**

| Page | Item |
| --- | --- |
| 2-3 | PRISMA Checklist |
| 4 | Table S1. Search Strategies |
| 5 | Figure S1. Leave-one-out sensitivity analysis for hypoglycemia. |
| 6 | Figure S2. Baujat plot for hypoglycemia. |
| 7 | Figure S3. Doi plot for hypoglycemia. |
| 7 | Figure S4. Leave-one-out sensitivity analysis for treatment success. |
| 8 | Figure S5. Baujat plot for treatment success. |
| 9 | Figure S6. Doi plot for treatment success. |
| 10 | Figure S7. Doi plot for recurrent hypoglycemia. |
| 11 | Figure S8. Funnel plot after trim and fill meta-analysis for recurrent hypoglycemia. |
| 11 | Figure S9. Leave-one-out sensitivity analysis for NICU admission. |
| 12 | Figure S10. Doi plot for NICU admission. |
| 13 | Figure S11. Doi plot for breastfeeding at discharge. |
| 14 | Figure S12. Doi plot for formula feeding. |
| 15 | Figure S13. Funnel plot after trim and fill meta-analysis for NICU admission. |
| 15 | Figure S14: Forest plot of recurrent hypoglycemia by trial purpose. |
| 16 | Figure S15: Forest plot of breastfeeding at discharge by trial purpose. |
| 16 | Figure S16: Forest plot of formula feeding by trial purpose. |
| 16 | Figure S17: Forest plot of NICU admission by trial purpose. |

**PRISMA checklist**

| **Section and Topic** | **Item #** | **Checklist item** | **Location where item is reported** |
| --- | --- | --- | --- |
| **TITLE** | | |  |
| Title | 1 | Identify the report as a systematic review. | 1 |
| **ABSTRACT** | | |  |
| Abstract | 2 | See the PRISMA 2020 for Abstracts checklist. | 3 |
| **INTRODUCTION** | | |  |
| Rationale | 3 | Describe the rationale for the review in the context of existing knowledge. | 4 |
| Objectives | 4 | Provide an explicit statement of the objective(s) or question(s) the review addresses. | 4 |
| **METHODS** | | |  |
| Eligibility criteria | 5 | Specify the inclusion and exclusion criteria for the review and how studies were grouped for the syntheses. | 5-6 |
| Information sources | 6 | Specify all databases, registers, websites, organisations, reference lists and other sources searched or consulted to identify studies. Specify the date when each source was last searched or consulted. | 5 |
| Search strategy | 7 | Present the full search strategies for all databases, registers and websites, including any filters and limits used. | 5 |
| Selection process | 8 | Specify the methods used to decide whether a study met the inclusion criteria of the review, including how many reviewers screened each record and each report retrieved, whether they worked independently, and if applicable, details of automation tools used in the process. | 5 |
| Data collection process | 9 | Specify the methods used to collect data from reports, including how many reviewers collected data from each report, whether they worked independently, any processes for obtaining or confirming data from study investigators, and if applicable, details of automation tools used in the process. | 5-6 |
| Data items | 10a | List and define all outcomes for which data were sought. Specify whether all results that were compatible with each outcome domain in each study were sought (e.g. for all measures, time points, analyses), and if not, the methods used to decide which results to collect. | 5-6 |
|  | 10b | List and define all other variables for which data were sought (e.g. participant and intervention characteristics, funding sources). Describe any assumptions made about any missing or unclear information. | 5-6 |
| Study risk of bias assessment | 11 | Specify the methods used to assess risk of bias in the included studies, including details of the tool(s) used, how many reviewers assessed each study and whether they worked independently, and if applicable, details of automation tools used in the process. | 6 |
| Effect measures | 12 | Specify for each outcome the effect measure(s) (e.g. risk ratio, mean difference) used in the synthesis or presentation of results. | 6 |
| Synthesis methods | 13a | Describe the processes used to decide which studies were eligible for each synthesis (e.g. tabulating the study intervention characteristics and comparing against the planned groups for each synthesis (item #5)). | 5-6 |
|  | 13b | Describe any methods required to prepare the data for presentation or synthesis, such as handling of missing summary statistics, or data conversions. |  |
|  | 13c | Describe any methods used to tabulate or visually display results of individual studies and syntheses. | 6 |
|  | 13d | Describe any methods used to synthesize results and provide a rationale for the choice(s). If meta-analysis was performed, describe the model(s), method(s) to identify the presence and extent of statistical heterogeneity, and software package(s) used. | 6 |
|  | 13e | Describe any methods used to explore possible causes of heterogeneity among study results (e.g. subgroup analysis, meta-regression). | 6 |
|  | 13f | Describe any sensitivity analyses conducted to assess robustness of the synthesized results. | 6 |
| Reporting bias assessment | 14 | Describe any methods used to assess risk of bias due to missing results in a synthesis (arising from reporting biases). | 6 |
| Certainty assessment | 15 | Describe any methods used to assess certainty (or confidence) in the body of evidence for an outcome. | 7 |
| **RESULTS** | | |  |
| Study selection | 16a | Describe the results of the search and selection process, from the number of records identified in the search to the number of studies included in the review, ideally using a flow diagram. | 6-7 |
|  | 16b | Cite studies that might appear to meet the inclusion criteria, but which were excluded, and explain why they were excluded. |  |
| Study characteristics | 17 | Cite each included study and present its characteristics. | 7,16-22 |
| Risk of bias in studies | 18 | Present assessments of risk of bias for each included study. | 7 |
| Results of individual studies | 19 | For all outcomes, present, for each study: (a) summary statistics for each group (where appropriate) and (b) an effect estimate and its precision (e.g. confidence/credible interval), ideally using structured tables or plots. | 7-8 |
| Results of syntheses | 20a | For each synthesis, briefly summarise the characteristics and risk of bias among contributing studies. | 7-8 |
|  | 20b | Present results of all statistical syntheses conducted. If meta-analysis was done, present for each the summary estimate and its precision (e.g. confidence/credible interval) and measures of statistical heterogeneity. If comparing groups, describe the direction of the effect. | 7-8 |
|  | 20c | Present results of all investigations of possible causes of heterogeneity among study results. | 7-8 |
|  | 20d | Present results of all sensitivity analyses conducted to assess the robustness of the synthesized results. | 7-8 |
| Reporting biases | 21 | Present assessments of risk of bias due to missing results (arising from reporting biases) for each synthesis assessed. | 7-8 |
| Certainty of evidence | 22 | Present assessments of certainty (or confidence) in the body of evidence for each outcome assessed. | 8 |
| **DISCUSSION** | | |  |
| Discussion | 23a | Provide a general interpretation of the results in the context of other evidence. | 9-10 |
|  | 23b | Discuss any limitations of the evidence included in the review. | 10-11 |
|  | 23c | Discuss any limitations of the review processes used. | 11 |
|  | 23d | Discuss implications of the results for practice, policy, and future research. | 11 |
| **OTHER INFORMATION** | | |  |
| Registration and protocol | 24a | Provide registration information for the review, including register name and registration number, or state that the review was not registered. | 5 |
|  | 24b | Indicate where the review protocol can be accessed, or state that a protocol was not prepared. | 5 |
|  | 24c | Describe and explain any amendments to information provided at registration or in the protocol. | 5 |
| Support | 25 | Describe sources of financial or non-financial support for the review, and the role of the funders or sponsors in the review. | 12 |
| Competing interests | 26 | Declare any competing interests of review authors. | 12 |
| Availability of data, code and other materials | 27 | Report which of the following are publicly available and where they can be found: template data collection forms; data extracted from included studies; data used for all analyses; analytic code; any other materials used in the review. | 12 |

Table S1. Search strategies

| Databases | Search strategies | Results | Limitations | Date of search |
| --- | --- | --- | --- | --- |
| PubMed | ( ("Hypoglycemia"[Mesh] OR hypoglycem*[tiab] OR hypoglycaem*[tiab] OR "low blood glucose"[tiab] OR "low glucose"[tiab]) AND ("Infant, Newborn"[Mesh] OR neonat*[tiab] OR newborn*[tiab] OR "new born"[tiab] OR "new-born"[tiab] OR infant*[tiab] OR baby[tiab] OR babies[tiab] OR preterm[tiab] OR premature[tiab]) AND ( ( ("Dextrose"[Mesh] OR "Glucose"[Mesh] OR dextrose[tiab] OR glucose[tiab]) AND (gel[tiab] OR gels[tiab] OR oral[tiab] OR buccal[tiab] OR mucosal[tiab]) ) OR "oral dextrose gel"[tiab] OR "oral glucose gel"[tiab] OR "buccal dextrose gel"[tiab] OR "buccal glucose gel"[tiab] OR "40% dextrose gel"[tiab] OR "40% glucose gel"[tiab] ) ) AND ( randomized controlled trial[pt] OR controlled clinical trial[pt] OR random*[tiab] OR placebo[tiab] OR trial[tiab] OR groups[tiab] ) NOT (animals[mh] NOT humans[mh]) | 267 | No limitations applied | 26 March 2026 |
| WOS | TS=( ( hypoglycem* OR hypoglycaem* OR "low blood glucose" OR "low glucose" ) AND ( neonat* OR newborn* OR "new born" OR "new-born" OR infant* OR baby OR babies OR preterm OR premature ) AND ( ((dextrose OR glucose) NEAR/2 gel*) OR "oral dextrose gel" OR "oral glucose gel" OR "buccal dextrose gel" OR "buccal glucose gel" OR ((dextrose OR glucose) NEAR/3 (oral OR buccal OR mucosal)) ) AND ( random* OR placebo OR trial OR "controlled trial" OR "clinical trial" ) ) | 406 | No limitations applied | 26 March 2026 |
| Cochrane CENTRAL | ( [mh Hypoglycemia] OR hypoglycem*:ti,ab,kw OR hypoglycaem*:ti,ab,kw OR "low blood glucose":ti,ab,kw OR "low glucose":ti,ab,kw ) AND ( [mh "Infant, Newborn"] OR neonat*:ti,ab,kw OR newborn*:ti,ab,kw OR "new born":ti,ab,kw OR "new-born":ti,ab,kw OR infant*:ti,ab,kw OR baby:ti,ab,kw OR babies:ti,ab,kw OR preterm:ti,ab,kw OR premature:ti,ab,kw ) AND ( ( ( [mh Dextrose] OR [mh Glucose] OR dextrose:ti,ab,kw OR glucose:ti,ab,kw ) AND ( gel*:ti,ab,kw OR oral:ti,ab,kw OR buccal:ti,ab,kw OR mucosal:ti,ab,kw ) ) OR "oral dextrose gel":ti,ab,kw OR "oral glucose gel":ti,ab,kw OR "buccal dextrose gel":ti,ab,kw OR "buccal glucose gel":ti,ab,kw OR "40% dextrose gel":ti,ab,kw OR "40% glucose gel":ti,ab,kw ) | 62 | Title Abstract Keyword | 26 March 2026 |
| Scopus | TITLE-ABS-KEY( ( hypoglycem* OR hypoglycaem* OR "low blood glucose" OR "low glucose" ) AND ( neonat* OR newborn* OR "new born" OR "new-born" OR infant* OR baby OR babies OR preterm OR premature ) AND ( ((dextrose OR glucose) W/2 gel*) OR "oral dextrose gel" OR "oral glucose gel" OR "buccal dextrose gel" OR "buccal glucose gel" OR ((dextrose OR glucose) W/3 (oral OR buccal OR mucosal)) ) AND ( random* OR placebo OR trial OR "controlled trial" OR "clinical trial" ) ) | 341 | title, Abstract, Keywords | 26 March 2026 |
| Embase | ( 'hypoglycemia'/exp OR hypoglycem*:ti,ab OR hypoglycaem*:ti,ab OR 'low blood glucose':ti,ab OR 'low glucose':ti,ab ) AND ( 'newborn'/exp OR 'newborn infant'/exp OR neonat*:ti,ab OR newborn*:ti,ab OR 'new born':ti,ab OR 'new-born':ti,ab OR infant*:ti,ab OR baby:ti,ab OR babies:ti,ab OR preterm:ti,ab OR premature:ti,ab ) AND ( ( ( 'dextrose'/exp OR 'glucose'/exp OR dextrose:ti,ab OR glucose:ti,ab ) AND ( gel*:ti,ab OR oral:ti,ab OR buccal:ti,ab OR mucosal:ti,ab ) ) OR 'oral dextrose gel':ti,ab OR 'oral glucose gel':ti,ab OR 'buccal dextrose gel':ti,ab OR 'buccal glucose gel':ti,ab OR '40% dextrose gel':ti,ab OR '40% glucose gel':ti,ab ) AND ( 'randomized controlled trial'/exp OR 'clinical trial'/exp OR random*:ti,ab OR placebo:ti,ab OR trial:ti,ab ) AND [humans]/lim | 256 | No limitations applied | 26 March 2026 |
| Total |  | 1332 |  |  |

Figure S1: Leave-one-out sensitivity analysis for hypoglycemia.

Figure S2: Baujat plot for hypoglycemia.

Figure S3: Doi plot for hypoglycemia

Figure S4: Leave-one-out sensitivity analysis for treatment success.

Figure S5: Baujat plot for treatment success.

Figure S6: Doi plot for treatment success

Figure S7: Doi plot for recurrent hypoglycemia

Figure S8: Funnel plot after trim and fill meta-analysis for recurrent hypoglycemia

Figure S9: Leave-one-out sensitivity analysis for NICU admission.

Figure S10: Doi plot for NICU admission

Figure S11: Doi plot for breastfeeding at discharge

Figure S12: Doi plot for formula feeding

Figure S13: Funnel plot after trim and fill meta-analysis for NICU admission

Figure S14: Forest plot of recurrent hypoglycemia by trial purpose.

Figure S15: Forest plot of breastfeeding at discharge by trial purpose.

Figure S16: Forest plot of formula feeding by trial purpose.

Figure S17: Forest plot of NICU admission by trial purpose.
